# Older Adults’ Emotion Recognition Ability Is Unaffected by Stereotype Threat

**DOI:** 10.3389/fpsyg.2020.605724

**Published:** 2021-01-07

**Authors:** Lianne Atkinson, Janice E. Murray, Jamin Halberstadt

**Affiliations:** Department of Psychology, University of Otago, Dunedin, New Zealand

**Keywords:** stereotype threat, older adults, young adults, ageing, cognition, social

## Abstract

Eliciting negative stereotypes about ageing commonly results in worse performance on many physical, memory, and cognitive tasks in adults aged over 65. The current studies explored the potential effect of this “stereotype threat” phenomenon on older adults’ emotion recognition, a cognitive ability that has been demonstrated to decline with age. In Study 1, stereotypes about emotion recognition ability across the lifespan were established. In Study 2, these stereotypes were utilised in a stereotype threat manipulation that framed an emotion recognition task as assessing either cognitive ability (stereotypically believed to worsen with age), social ability (believed to be stable across lifespan), or general abilities (control). Participants then completed an emotion recognition task in which they labelled dynamic expressions of negative and positive emotions. Self-reported threat concerns were also measured. Framing an emotion recognition task as assessing cognitive ability significantly heightened older adults’ (but not younger adults’) reports of stereotype threat concerns. Despite this, older adults’ emotion recognition performance was unaffected. Unlike other cognitive abilities, recognising facially expressed emotions may be unaffected by stereotype threat, possibly because emotion recognition is automatic, making it less susceptible to the cognitive load that stereotype threat produces.

## Introduction

Reminding an individual of negative stereotypes about a group to which they belong can lead to concerns about confirming these negative stereotypes, and, in turn, to performance deficits on stereotype-relevant tasks (Steele and Aronson, 1995; Lamont et al., 2015; Spencer et al., 2016). For example, subtly evoking gender stereotypes can cause women to underperform on mathematics tasks, on which they are stereotypically expected to perform worse than men (Doyle and Voyer, 2016). Similarly, eliciting negative stereotypes about African Americans’ academic abilities leads to underperformance on academic tests (Nguyen and Ryan, 2008; Spencer et al., 2016). This well-documented phenomenon is known as *stereotype threat*.

One group frequently subject to prejudice and age-related stereotypes is older adults. Meta-analyses have consistently found that people hold more negative attitudes toward older people than younger people (Kite and Johnson, 1988; Kite et al., 2005; North and Fiske, 2012). Commonly held negative attitudes and stereotypes include the beliefs that attractiveness declines with age, that older adults are less competent than young adults, that older adults lack creativity, and that older adults are less able than young people to learn new skills (Hummert et al., 1994; Kite et al., 2005; Swift et al., 2013).

As with race- and gender-based stereotypes, evoking negative stereotypes about ageing can impair older adults’ performance on stereotype-relevant tasks, including memory, cognitive, and physical tasks (Lamont et al., 2015). In one study, for example, Desrichard and Köpetz (2005) asked young and older adults to complete a “running an errand” task (Radziszewska and Rogoff, 1991), which involved memorising a list of shop items and then, without viewing the list, using a map to work out the quickest way of getting from shop to shop to purchase each item. Before completing this task, participants were either told that the task relies on memory skills (which are stereotypically expected to decline with age), or that it relies on orientation skills. Older adults’ performance on the errand task was much poorer when it was framed as a memory task, compared to when it was framed as an orientation task. In contrast, young adults’ performance did not differ between the two conditions. These results suggested that making the task relevant to stereotypes about age-related memory decline led to feelings of stereotype threat in older adults, which subsequently further impaired their memory performance.

Researchers have yet to come to a consensus about the underlying mechanisms of stereotype threat effects. The cognitive load hypothesis (Schmader et al., 2008) purports that eliciting stereotypes about a target group leads its members to feel highly motivated to disconfirm the stereotype. Consequently, working memory and other cognitive resources are preferentially devoted to avoiding failure on the task and regulating feelings of frustration, which interrupts an individual’s ability to complete the test items successfully (Schmader et al., 2008). This hypothesis has been supported by various studies demonstrating that stereotype threat causes reductions in working memory capacity and other executive functions (e.g., Rydell et al., 2014) and increases in mental load (e.g., Croizet et al., 2004) in young adults. However, what is currently unclear is the extent to which this mechanism also underlies older adults’ experience of stereotype threat.

Another possible explanation of stereotype threat in older adults is incompatible regulatory focus (Higgins, 1997; Barber, 2017). According to regulatory focus theory, different tasks require different motivational orientations (Crowe and Higgins, 1997; Higgins, 1997). Specifically, a “promotion focus” emphasises achieving gains and is more compatible with tasks framed in terms of rewards, whereas a “prevention focus” emphasises avoiding losses and is more compatible with tasks framed in terms of losses (Crowe and Higgins, 1997; Barber et al., 2015). Adopting a focus that is incompatible (versus compatible) with a task can lead to poor performance (Higgins, 2000; Barber and Mather, 2013). According to Barber (2017), age-related stereotype threat effects can be understood as focus-task incompatibilities, in that cognitive tasks (e.g., recalling as many items as possible in a memory test) are generally promotion focussed, whereas stereotype threat is generally prevention focussed, inducing a motivation to avoid poor performance.

Regardless of its underlying mechanism, however, stereotype threat has been shown to reliably impair older adults’ cognitive abilities (Lamont et al., 2015). A meta-analysis of 32 published and unpublished articles demonstrated a robust effect of age-related stereotype threat on older adults’ performance on a number of tasks, including mathematics, letter cancellation, mental rotation, and memory (Lamont et al., 2015). Another cognitive ability that might also be susceptible to age-related stereotype threat is emotion recognition. Emotion recognition is a socially relevant set of skills that involves perceiving, evaluating, recognising, and labelling emotional expressions (Adolphs, 2002). These integrated cognitive processes are thought to rely on specific regions of the brain, including the occipital cortex, fusiform cortex, amygdala, insula, and somatosensory cortex (Adolphs, 2002; Vuilleumier and Pourtois, 2007; Van de Riet et al., 2009). Although emotion has often been conceptualised as a separate and distinct process to cognition, many researchers now argue that affect and cognition are highly integrated in the brain and should be conceptualised as one system (Pessoa, 2008; Hoemann and Barrett, 2019).

Notably, older adults (aged over 60 years) have consistently been shown to be less accurate than young adults at recognising emotions across a number of modalities, including facial expressions, body language, and tone of voice (Ruffman et al., 2008; Gonçalves et al., 2018; Hayes et al., 2020). In particular, older adults are significantly worse than young adults at recognising anger, fear, sadness, disgust, and (depending on the emotion stimuli used) happiness and surprise (Ruffman et al., 2008; Gonçalves et al., 2018; Hayes et al., 2020). Such deficits in emotion recognition ability may contribute to older adults experiencing difficulties in other areas of social functioning, including discriminating between appropriate and inappropriate behaviour (i.e., faux pas; Halberstadt et al., 2011), and inferring what another person is truly feeling (Ruffman et al., 2019).

Although the exact causes of the apparent age-related decline in emotion recognition remain inconclusive, several theories have been proposed. One prominent idea is derived from the socioemotional selectivity theory, which posits that older adults may direct their attention to positive and emotionally meaningful pursuits and stimuli in order to regulate their emotions and obtain the most enjoyment from the time remaining in their lives (Carstensen et al., 2003). This “positivity effect” may lead older adults to reappraise negative emotions expressed by another individual in a more positive light, in order to effectively regulate their own emotions (Mather and Carstensen, 2003; Ruffman et al., 2008). In doing so, they may incorrectly interpret and categorise negative emotions (Ruffman et al., 2008). Other theories purport that age-related declines in emotion recognition ability result from specific changes in the brain and/or the general cognitive decline that occurs with age (see Ruffman et al., 2008 for a review).

It is also possible, however, that age-related differences in emotion recognition have been caused or exaggerated by stereotype threat. Simply using task instructions that emphasise abilities that decline with age (Rahhal et al., 2001), or informing older participants that they are being compared with younger adults (Abrams et al., 2006), has been shown to produce stereotype threat effects on older adults’ cognition. Thus, studies that have compared young and older adults’ ability to recognise emotions (and have informed their participants of this) may have unknowingly evoked feelings of stereotype threat in older adults, which could have artefactually reduced their performance on emotion recognition tasks.

The current research aimed to investigate whether older adults’ emotion recognition, like memory and other cognitive abilities that decline with age, is impaired by age-related stereotype threat. Two studies were conducted to answer this question. Study 1 examined the stereotypes of older adults across a range of abilities, including emotion recognition. Study 2 then presented an emotion recognition task to young and older adults, manipulating the framing of the task. To clearly demonstrate that any impact of age-related stereotype threat on emotion recognition is not a generalised effect, we framed the emotion task as requiring cognitive ability or social ability, and based on the results of Study 1, predicted that older adults’ (but not young adults’) perceived stereotype threat and emotion recognition performance would differ as a function of task framing. Specifically, it was hypothesised that framing emotion recognition as a cognitive ability (which Study 1 participants believed declines with age), would evoke feelings of stereotype threat in older but not young adults, and, in turn, cause a decline in older adults’ emotion recognition ability. In contrast, we predicted that framing emotion recognition as a social ability (which Study 1 participants believed remains intact with age) would neither threaten nor impair older adults.

## Study 1

There were two primary objectives of Study 1. First, we wanted to examine the stereotypes of older adults with regard to emotion recognition and other cognitive and social skills. Participants were asked to consider the relative competence of a typical 25-year-old adult and a typical 75-year-old adult in a number of different task domains (such as memory, emotion recognition, cognitive ability, and social skills). Whereas older adults are usually considered less competent than young adults with regard to cognitive functioning (Prohaska et al., 1984; Singer, 1986; Swift et al., 2013), they are often expected to be relatively competent in certain social domains, such as being able to settle arguments, being polite, and understanding other people’s points of view (Swift et al., 2013). Second, Study 1 sought to determine whether lay people primarily conceptualise emotion recognition as a cognitive or a social task. Participants should theoretically be vulnerable to stereotype threat only if they hold a relevant stereotype in the domain; thus, older participants’ emotion recognition performance should only be impaired to the extent that they view emotion recognition as the kind of task on which older adults’ performance tends to decline.

Individuals from three different age groups were recruited to participate in the current study. Young adults (aged 18–30) and older adults (aged 65 and over) were included in order to compare the perceptions about ageing between those who belong to the stigmatised age group and those who do not. A third participant age group (aged 50–64 years – classified in the current study as “middle-older” adults) was included for exploratory purposes, to obtain an idea of the current ageing stereotypes held by individuals who do not belong to the stigmatised age group, but are soon approaching it.

### Methods

#### Participants

Young, middle-older, and older adults responded to the current survey as part of a larger study. The participants included 123 young adults (55 male) aged 18–30 (*M* = 25.2 years, *SD* = 3.17 years), 154 middle-older adults (68 male) aged 50–64 (*M* = 55.7 years, *SD* = 3.83), and 143 older adults (61 male) aged 65–99 (*M* = 69.0 years, *SD* = 4.45 years) who reside in the United States. Participants were recruited through Amazon’s Mechanical Turk (MTurk), a crowdsourcing Internet marketplace that recruits “workers” to complete tasks for compensation. For this 20–30 min study, MTurk workers were remunerated $0.90 or $1.00^1^. All participants spoke English and self-reported that they had not experienced or were currently experiencing any neurological difficulties.

#### Procedure

After providing informed consent, participants were presented with a survey designed in and hosted by Qualtrics^2^, which asked a number of questions about their perceptions of young and older adults’ competencies in various task domains (see Supplementary Material). Following Swift et al. (2013), participants were asked to choose who they believed would perform better in particular skill areas: adults aged 25, adults aged 75, or whether they perceive there to be no difference in ability between these age groups. The original items used in Swift et al.’s (2013) study were retained (e.g., solving a crossword, looking after children, and settling arguments), and additional items were added to answer the current research question (i.e., completing cognitive tasks, social interaction, recognising emotions in other people’s faces, understanding how someone is feeling or what they are thinking, understanding others’ emotional body language, and recognising the emotion in others’ tone of voice). Some additional filler items (e.g., reading for pleasure and completing a running race) were added to prevent the research question being obvious to participants. After the participants completed these items, they were presented with the following forced-choice question: “Do you think the recognition of emotions in other people’s faces is predominantly a cognitive task? OR a social task?” Participants were able to select only one of these responses.

### Results and Discussion

#### Data Analysis

The following data were analysed using chi-square tests of goodness of fit and a chi-square test of independence. For all chi-square tests, the sample size per cell was greater than five; therefore, the assumptions for using a chi-square test were met. In all cases, a test statistic was considered significant if the associated *p*-value was less than 0.05. Where the chi-square test of independence was utilised, Cramer’s *V* effect size is reported.

#### Beliefs About Young and Older Adults’ Competencies

With regard to participants’ beliefs about the competencies of a 25-year-old versus a 75-year-old adult, the percentage of participants who selected each response (“adults aged 25”, “no difference”, or “adults aged 75”) was calculated for each competency domain, separately for each age group. These percentages, for each competency domain and each age group, are given in full in Supplementary Table 1, along with associated chi-square tests of goodness-of-fit. The data for the competency domains that are integral to the present study’s research question are presented in Table 1.

**TABLE 1:** Percentage of participants who selected either “adults aged 25,” “no difference,” or “adults aged 75” as most competent in relevant domains, for each participant age group.

Competency domain	Participant age group	Percentage of participants who selected “adults aged 25”, “no difference” or “adults aged 75”		
		Adults aged 25	No difference	Adults aged 75
Social Interaction	18–30	**41.5**	**51.2**	7.3
	50–64	30.5	**54.5**	14.9
	65+	18.2	**67.1**	14.7
	Mean (all ages)	29.5	**57.9**	12.6
Recognising emotions in others’ faces	18–30	10.6	**56.1**	33.3
	50–64	5.2	**45.5**	**49.4**
	65+	4.2	**51.7**	**44.1**
	Mean (all ages)	6.4	**50.7**	**42.9**
Understanding how someone is feeling/what they are thinking	18–30	17.1	**59.3**	23.6
	50–64	3.2	**42.2**	**54.5**
	65+	4.2	**49.7**	**46.2**
	Mean (all ages)	7.6	**49.8**	**42.6**
Completing cognitive tasks (e.g., involving attention, problem-solving, and decision-making)	18–30	**69.1**	28.5	2.4
	50–64	**56.5**	37.7	5.8
	65+	**53.1**	**40.6**	6.3
	Mean (all ages)	**59.0**	36.0	5.0
Understanding others’ emotional body language	18–30	11.4	**68.3**	20.3
	50–64	11.7	**48.7**	**39.6**
	65+	4.2	**47.6**	**48.3**
	Mean (all ages)	9.0	**54.0**	36.9
Completing memory tasks	18–30	**84.6**	13.0	2.4
	50–64	**83.8**	13.6	2.6
	65+	**77.6**	19.6	2.8
	Mean (all ages)	**81.9**	15.5	2.6
Recognising the emotion in others’ tone of voice	18–30	9.8	**69.1**	21.1
	50–64	2.6	**55.2**	**42.2**
	65+	0	**55.2**	**44.8**
	Mean (all ages)	3.8	**59.3**	36.9

*Percentages presented in bold are the highest within each participant age group (significance level of *p* < 0.05). Where the second-highest percentage is statistically equivalent to the highest, that percentage is also bolded.*

Participants endorsed many age-related stereotypes that are consistent with stereotypes established in previous studies (e.g., Swift et al., 2013). For example, participants from all three age groups judged a typical 25-year-old adult to be more competent than a 75-year-old adult at driving, learning new skills, using the Internet, completing memory tasks, completing computer tasks, and completing a running race. Importantly, the majority of participants from all three age groups judged 25-year-olds to be more competent at completing cognitive tasks, compared to 75-year-olds. Regarding the domain of social interaction, a majority of young, middle-older, and older adults judged there to be no difference in competency between adults aged 25 and adults aged 75.

Regarding the specific research question concerning emotion recognition, participants judged a typical 75-year-old individual to be equal to, or more competent than, a typical 25-year-old in every task domain related to recognising emotions. Specifically, when considering an individual’s ability to recognise emotions in other people’s faces, to understand how someone is feeling or what they are thinking, to understand other people’s emotional body language, and to recognise the emotion in somebody’s tone of voice, participants across all age groups judged that there is no difference in competency between adults aged 25 and adults aged 75, or that adults aged 75 are *more* competent. In contrast to age-related stereotypes about memory difficulties, cognitive decline, and deterioration in physical ability, and despite an abundance of laboratory-based research demonstrating that emotion recognition declines with age (Ruffman et al., 2008; Gonçalves et al., 2018; Hayes et al., 2020), participants, regardless of age, reported that older adults are equal to or better than young adults at recognising emotions.

#### Perceptions of Emotion Recognition as a Cognitive or Social Task

Participants’ perceptions of emotion recognition as either a cognitive task or a social task were related to participant age group, according to a chi-square test of independence, *X*^2^ (2, *N* = 420) = 8.01, *p* = 0.02, *V* = 0.138. Follow-up chi-square tests of goodness of fit indicated that a greater percentage of young participants (67.5%) consider the recognition of emotions to be a social task as opposed to a cognitive task, *X*^2^ (1, *N* = 123) = 15.03, *p* < 0.001. For the middle-older participants, the chi-square test did not reach significance, *X*^2^ (1, *N* = 154) = 3.14, *p* = 0.08, indicating that a relatively equal percentage of middle-older adults consider the recognition of emotions to be a social task (57.1%) versus a cognitive task (42.9%). Similarly, an equal percentage of older participants consider emotion recognition to be a social task (50.3%) versus a cognitive task (49.7%), *X*^2^ (1, *N* = 143) = 0.01, *p* = 0.93.

In sum, the current study showed that, whereas lay people believe older adults to be as good as young adults at social interaction, they believe older adults to be inferior on cognitive tasks. It also indicated that middle-older and older adults were equally likely to consider emotion recognition to be a cognitive or a social task, whereas young adults were more likely to view it as a social task. Therefore, framing an emotion recognition task as assessing cognitive ability, rather than social ability, could conceivably produce stereotype threat effects in older adults. This was tested in Study 2.

## Study 2

Young (aged 18–30 years) and older (aged over 65 years) adults were assigned to one of three task framing conditions: cognitive, social, or control, in which the emotion recognition task was described as assessing cognitive, social, or general abilities, respectively. Inclusion of the social task framing condition allowed for a stereotype-specific control condition. Participants completed the Geneva Emotion Recognition Test – Short Version (GERT-S; Schlegel and Scherer, 2016). Subsequently, participants answered two explicit questions about how stereotype-threatened they had felt while completing the test. We hypothesised that, due to prevalent stereotypes about cognitive decline with age, and based on the results of Study 1, older adults would report being more threatened in the condition in which emotion recognition was framed as a cognitive task, compared to the social and general ability control conditions. As a result of this stereotype threat, it was expected that older adults would experience a performance deficit on the emotion recognition task in the cognitive condition, relative to the other two conditions. Conversely, it was hypothesised that young adults’ reported level of stereotype threat and performance on the GERT-S would remain constant across the three conditions. In other words, we predicted an interaction effect such that older adults’ (but not young adults’) perceived stereotype threat and performance on the emotion recognition task would differ as a function of stereotype threat condition.

### Methods

#### Participants

The participants were 122 young adults (68 male) aged 18–30 (*M* = 25.82 years, *SD* = 3.05 years) and 117 older adults (52 male) aged 65–88 (*M* = 69.47 years, *SD* = 4.44 years) from the United States. Amazon Mechanical Turk was used to recruit participants, who were compensated with $0.90 or $1.00. All participants self-reported being proficient in English and self-reported that they had not experienced dementia or other neurological difficulties. Participants completed the current experiment as part of a larger study.

#### Stimuli and Measures

##### The geneva emotion recognition test – short version (GERT-S)

The GERT-S (Schlegel and Scherer, 2016) is a 42-item emotion recognition task designed to assess people’s ability to recognise emotions in another person’s facial expression, tone of voice, and body language. On average, the GERT-S takes approximately 10 min to complete. Each trial consists of a short video clip of an actor portraying an emotion, with nonsensical syllables used to express emotional tone of voice. The video clips used are from the Geneva Multimodal Emotion Portrayals database (GEMEP; Bänziger et al., 2012). Over the course of the test, 10 actors (five male, five female) convey 14 different emotions (pride, joy, amusement, pleasure, relief, interest, surprise, anxiety, fear, despair, sadness, disgust, irritation, and anger). Each emotion is presented three times in random order, resulting in a total of 42 trials. After each video clip, 14 emotion words were presented in a circular arrangement and participants were required to select the emotion label that they believed best described the emotion portrayed by the actor in the clip.

#### Procedure

Participants were randomly assigned to one of three conditions: cognitive, social, or control. In the cognitive condition, the recognition of emotions was framed as a task that assesses cognitive ability, with participants being told that “the purpose of this study is to examine people’s cognitive ability at different ages” and that young and older adults were being compared. In the social condition, participants were told that “the purpose of this study is to examine people’s social ability at different ages” and that young and older adults were being compared. Finally, in the control condition, they were told that “the purpose of this study is to examine people’s ability on various tasks”, and that different types of people would be taking part in the research (i.e., no mention of age group comparisons).

After carrying out the emotion recognition task, participants were asked 2 questions to assess their explicit stereotype threat-based concerns – “Were you worried that your ability to perform well on these tasks was affected by your age?” and “Were you worried that if you performed poorly on the test, the researcher would attribute your poor performance to your age?” (see Gaillard et al., 2011). Participants were required to respond using a scale from 1 (not at all) to 7 (very much).

### Results

#### Data Analysis

Data from the current study were primarily analysed using analysis of variance (ANOVA), with Huynh-Feldt corrected values reported if Mauchly’s Test showed the assumption of sphericity to be violated. Where necessary, significant interactions were further analysed using *post hoc* Bonferroni multiple comparisons in cases of three groups being compared (e.g., comparing scores between stereotype threat conditions) or *t*-tests when only two groups were being compared (e.g., comparing scores between young and older adults). Where *t-*tests were utilised, Levene’s Test for Equality of Variances was also employed, and corrections subsequently made for any unequal variances.

#### Threat-Based Concerns Across Conditions

A Pearson correlation coefficient demonstrated that participants’ responses on the 7-point scale for the two questions related to threat concerns were positively correlated, *r* = 0.66, *n* = 239, *p* < 0.001; therefore, responses for the two questions were averaged to create a single threat score for each participant. Participants’ threat scores were then examined in a 2 (participant age group: young, older) × 3 (condition: control, cognitive, social) ANOVA, revealing a significant main effect of condition, *F*(2,233) = 3.88, *p* = 0.02, η_*p*_^2^ = 0.03. Follow-up multiple comparisons using Bonferroni correction demonstrated that self-reported threat scores were significantly higher in the cognitive condition (*M* = 2.20, *SD* = 1.60) compared to the control condition (*M* = 1.64, *SD* = 1.19), *p* = 0.02. Threat scores did not significantly differ between the social condition and the control condition, *p* = 0.76, nor between the cognitive condition and the social condition (*M* = 1.88, *SD* = 1.15), *p* = 0.40.

**FIGURE 1:**
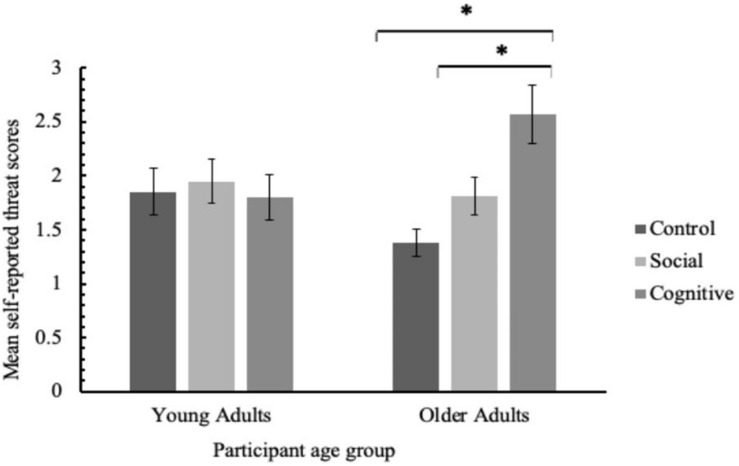
Mean self-reported threat scores as a function of participant age group and stereotype threat condition. Error bars denote one standard error around the mean and asterisks signify a significant difference in mean scores between conditions.

As predicted, there was also a significant interaction, depicted in Figure 1, between condition and participant age group, *F*(2,233) = 4.85, *p* = 0.009, η_*p*_^2^ = 0.04, which was explored with one-way ANOVAs on young and older participants’ data separately. For young adults, there was no main effect of condition on threat scores, *F*(2,119) = 0.11, *p* = 0.90, η_*p*_^2^ = 0.00. However, for older adults, the effect of condition on threat scores was significant, *F*(2,114) = 8.95, *p* < 0.001, η_*p*_^2^ = 0.14. Bonferroni comparisons established that older adults were significantly more threatened in the cognitive condition (*M* = 2.57, *SD* = 1.74) than both the social (*M* = 1.81, *SD* = 1.05), *p* = 0.028, and control conditions (*M* = 1.38, *SD* = 0.74), *p* < 0.001. There was no significant difference in self-reported threat between the social and control conditions, *p* = 0.45.

#### Effect of Stereotype Threat Condition on Young and Older Adults’ Emotion Recognition Accuracy

**FIGURE 2:**
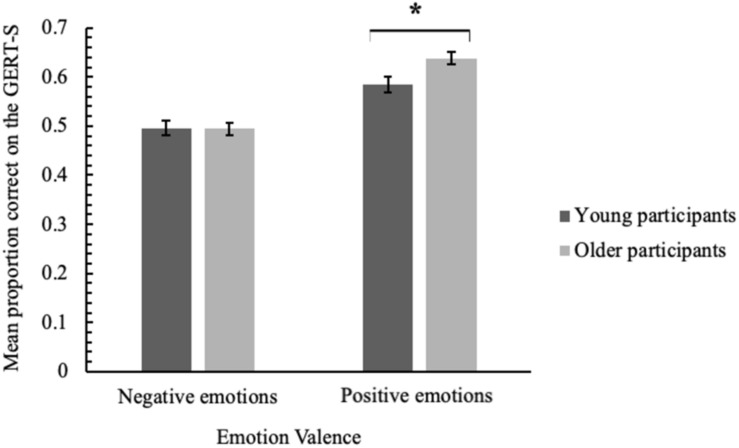
Mean proportion correct on the GERT-S for negative and positive emotions, as a function of participant age group. Error bars denote one standard error around the mean and the asterisk signifies a significant difference between participant age group means.

Separate negative and positive GERT-S scores were generated for each participant by calculating the respective proportion of negative emotions (anxiety, fear, despair, sadness, disgust, irritation, and anger) and positive emotions (pride, joy, amusement, pleasure, surprise, interest, and relief) that they correctly labelled^3^. GERT-S scores were then analysed in a 2 (emotion valence: negative, positive) × 2 (participant age group: young, older) × 3 (condition: control, cognitive, social) mixed ANOVA. A significant main effect of emotion valence indicated that participants were poorer at correctly labelling negative emotions (*M* = 0.50, *SD* = 0.15) than positive emotions (*M* = 0.61, *SD* = 0.16), *F*(1,233) = 126.41, *p* < 0.001, η_*p*_^2^ = 0.35. This effect was qualified by an interaction with age group, *F*(1,233) = 6.54, *p* = 0.01, η_*p*_^2^ = 0.03, displayed in Figure 2. For negative emotions, GERT-S scores did not significantly differ between young adults (*M* = 0.50, *SD* = 0.16) and older adults (*M* = 0.49, *SD* = 0.14), *t*(237) = 0.12, *p* = 0.90, but older adults (*M* = 0.64, *SD* = 0.13) were more accurate than young adults (*M* = 0.58, *SD* = 0.17) at recognising positive emotions *t*(223.4) = 2.71, *p* = 0.007.

Importantly for the current research hypotheses, young and older adults’ ability to recognise emotions did not vary as a function of whether they were told that the task was assessing general ability, social ability, or cognitive ability; there were no significant effects of stereotype threat condition on GERT-S scores (all *p*s > 0.05; see Table 2 for all ANOVA results including stereotype threat condition as a variable).

**TABLE 2:** Analysis of variance (ANOVA) results for main and interaction effects on GERT-S accuracy scores (proportion correct) involving stereotype threat condition.

Source	*df*	*MS*	*F*	*p*	η _*p*_^2^
Condition	2	0.026	0.780	0.460	0.007
Participant Age*Condition	2	0.020	0.588	0.556	0.005
Emotion Valence*Condition	2	0.034	2.70	0.069	0.023
Emotion Valence*Participant Age*Condition	2	0.008	0.618	0.540	0.005

### Discussion

When emotion recognition was framed as a task that assesses cognitive ability – believed to decline with age – older participants reported heightened levels of stereotype threat, compared to the control condition and social condition. Young participants’ self-reported threat did not differ between experimental conditions. However, despite older adults’ heightened threat concerns, their performance on the emotion recognition task was unaffected. The latter finding departs from earlier studies demonstrating that stereotype threat negatively affects older adults’ performance on several cognitive tasks (Lamont et al., 2015). Emotion recognition may be one cognitive ability that is resilient against stereotype threat.

A secondary finding was that, contrary to many previous studies and meta-analyses (Ruffman et al., 2008), older adults were equally competent as young adults at recognising negative emotions and better at recognising positive emotions. This finding is consistent with studies showing that older adults’ recognition of dynamic – as opposed to static – expressions of positive emotions may, in fact, remain intact (Murphy et al., 2010). For example, Murphy et al. (2010) found that, in one study, older adults did just as well as young adults at distinguishing between posed and spontaneous dynamic smiles, and in a second study, actually performed better than young adults. Their findings, along with the present study’s findings, appear to support the idea that employing dynamic emotion stimuli may improve older adults’ recognition of emotions – in particular, positive emotions (Ruffman, 2011). Compared to static images, dynamic emotion stimuli contain increased contextual information, which has been shown to be more important to the processing of facial emotions for older adults than for younger adults (Richter et al., 2010; Noh and Isaacowitz, 2013). The finding that older adults exhibited better recognition of positive emotions compared to negative emotions is also consistent with socioemotional selectivity theory (e.g., Carstensen, 2006), which assumes greater motivation to attend to, and in turn to recall accurately, positive (versus negative) information.

Of note, a recent meta-analysis by Hayes et al. (2020) demonstrated that older adults were worse than young adults at recognising emotions (negative and positive) even when video stimuli were used. However, Hayes et al.’s (2020) meta-analysis focussed on facial emotion recognition alone, and therefore did not include studies that employed multimodal emotion stimuli (i.e., audio-visual portrayals of emotion that incorporates face, voice, and body). Therefore, it is possible that stimulus sets comprised of dynamic, multimodal emotion displays (such as the GERT-S used in the current study) provide a greater amount of contextual information and are more ecologically valid than dynamic facial emotion displays and, in turn, may advantage older adults.

## General Discussion

The main finding of the current studies is that when emotion recognition is framed as assessing cognitive ability (widely believed to decline with age), older adults, but not young adults, report feeling significantly more threatened compared to when the task is framed as assessing social or general abilities. This finding is consistent with previous studies in which there were main or interaction effects of age-related stereotype threat (pertaining to cognitive or other stereotype-relevant tasks) on older adults’ self-reported perceived threat (e.g., Kang and Chasteen, 2009; Gaillard et al., 2011; Swift et al., 2013; Barber et al., 2015). However, despite older adults’ feelings of threat being heightened by the stereotype threat manipulation, their performance on the emotion recognition task was not affected.

One account of previously demonstrated effects of stereotypes on cognitive task performance is provided by the cognitive load hypothesis, which posits that stereotype threat leads an individual to turn their focus toward the stereotype, thus increasing distracting thoughts (Schmader et al., 2008). In theory, the resulting increase in cognitive load reduces the amount of cognitive resources that can be applied to the stereotype-relevant task, subsequently impairing task performance (Schmader et al., 2008). Previous work suggests that stereotype threat may be effective only when controlled processing is engaged. Mazerolle et al. (2012) investigated the effects of stereotype threat on the automatic and controlled aspects of a memory recall task and found that age-related stereotype threat only impaired older adults’ controlled use of memory, and actually improved their automatic recall. Relatedly, Eich et al. (2014) found that stereotype threat negatively affected controlled retrieval but did not affect item encoding. Thus, one possible account of the current results is that, compared to other cognitive tasks, recognising emotions engages automatic processes rather than controlled processes, making emotion recognition less susceptible to the effects of increased cognitive load.

A number of studies have supported the idea that the recognition of emotions – and the integration of emotions with the environment – largely involves automatic, reflexive, and effortless processes that require few mental resources and are less susceptible to negative effects from cognitive load (Tracy and Robins, 2008; Aviezer et al., 2011; Mumenthaler and Sander, 2015). Casares-Guillén et al. (2016), however, found that cognitive load reduced performance on emotion recognition tasks (a finding consistent with the view that emotion recognition engages controlled processes). Still others have found that recognition of emotions in unfamiliar faces may involve a combination of automatic, bottom-up processes and controlled, top-down processes (Yan et al., 2017). In sum, researchers have not yet come to a consensus about the automaticity of emotion processing, and although the present studies are consistent with the idea that emotion recognition is impervious to cognitive load caused by stereotype threat, they do not directly test this hypothesis.

Given that emotion recognition is a task that is likely to be most compatible with a promotion focus (Sassenrath et al., 2014), one might expect that older adults’ emotion recognition ability would be reduced by their adoption of a prevention focus, caused by stereotype threat. Instead, the current studies’ results demonstrated that emotion recognition was unaffected by feelings of stereotype threat, thus failing to provide evidence in support of the regulatory fit hypothesis. However, given that regulatory focus was neither tested nor experimentally manipulated in the current research, it should not be ruled out as an underlying mechanism of age-related stereotype threat.

The current research was limited, among other ways, by the neglect of potential moderating or mediating variables. Individual differences that have previously been shown to moderate effects of stereotype threat, such as coping sense of humour (Ford et al., 2004), denial of stereotypes (Von Hippel et al., 2005), and defensive pessimism (Perry and Skitka, 2009), could usefully be included in future research. Furthermore, future research could include additional variables that have been shown to mediate stereotype threat effects in some cases, such as anxiety (e.g., Tempel and Neumann, 2014; Lu et al., 2015). Another factor to consider is the use of MTurk to recruit both young and older participants. Generally, the limited existing research suggests that data obtained using MTurk samples are comparable to data obtained from the general population, including for older adults (e.g., Lemaster et al., 2015; Mortensen and Hughes, 2018). However, a very recent study found that, compared to a sample of the general older adult population, older adults recruited via MTurk have higher cognitive functioning, self-rated memory, and self-rated health (Ogletree and Katz, 2020). Thus, it is possible that the current studies’ use of participants from MTurk may limit the generalisability of the current findings to the general population.

Our findings have both methodological and applied implications. In terms of the methodology, they suggest that researchers can use recruitment advertisements that openly request older adults’ participation, or inform older adults that their emotion recognition abilities will be compared with young adults’, without fear that stereotype threat will artefactually worsen older adults’ emotion recognition ability. (Researchers may nevertheless want to avoid where possible using the term “cognitive” in their instructions, simply in the interest of participants’ comfort). From an applied perspective, the results suggest that interventions intended to reduce age-related stereotypes would be unlikely to improve older adults’ ability to recognise other people’s emotions. Conversely, older adults’ exposure to stereotypes outside the laboratory are also unlikely to impair their emotion recognition skills. This is especially encouraging in light of research demonstrating negative consequences of social deficits in older adults, such as greater cognitive decline (Seeman et al., 2001; Zunzunegui et al., 2003; Kelly et al., 2017; Evans et al., 2018), heightened risk of early mortality (Holt-Lunstad et al., 2015), and increased disability (Mendes de Leon et al., 2003).

In conclusion, the current research was the first to investigate whether older adults’ recognition of emotions, an ability known to decline with age, is impaired by age-related stereotype threat. Two studies provided evidence that emotion recognition may be one aspect of cognition that is unaffected by stereotype threat. This might be because emotion recognition involves more reflexive, automatic processes as opposed to deliberate, controlled processes, making it less susceptible to cognitive load increases produced by stereotype threat.

## Data Availability Statement

The raw data supporting the conclusions of this article will be made available by the authors, without undue reservation.

## Ethics Statement

The studies involving human participants were reviewed and approved by the University of Otago Human Ethics Committee. The participants provided their written informed consent to participate in this study.

## Author Contributions

LA, JM, and JH designed the studies and interpreted the data analyses. LA completed data collection, analysed the data, and wrote the first draft of the manuscript. All authors wrote sections of the manuscript, revised the manuscript critically, and approved the final submission.

## Conflict of Interest

The authors declare that the research was conducted in the absence of any commercial or financial relationships that could be construed as a potential conflict of interest.

## Footnotes

1 Remuneration was increased slightly partway through the study to better incentivise participation.

2 https://www.qualtrics.com

3 In line with the advice of Schlegel and Scherer (2016), recognition of discrete emotions was not analysed due to the small number of items (three) per emotion in the GERT-S. However, given the potential role of the positivity effect (Carstensen, 2006) in older adults’ recognition of emotions, it was considered important to include emotion valence as a within-subjects variable.
