# Supplementary material for: Older Adults’ Emotion Recognition Ability Is Unaffected by Stereotype Threat
Source: Front Psychol. 2021 Jan 7;11:605724. doi: 10.3389/fpsyg.2020.605724 (PMC7817847; doi:10.3389/fpsyg.2020.605724)
Supplement: Supplementary file 1 [file Data_Sheet_1.PDF]

Supplementary Material

1 Questions About Age-related Stereotypes Asked in Study 1

Please consider the following areas of competence. For each domain, please indicate the group that you think would be more competent: adults aged 25, adults aged 75, or both would perform equally well.

1. Solving crossword puzzles

2. Being polite

3. Driving

4. Social interaction*

5. Understanding others' viewpoints*

6. Learning new skills

7. Using the internet*

8. Settling arguments

9. Making financial decisions

10. Recognising emotions in others' faces*

11. Being creative

12. Understanding how someone is feeling or what they are thinking*

13. Reading for pleasure*

14. Completing cognitive tasks (e.g., involving attention, problem-solving, and decision-making)*

15. Understanding others' emotional body language*

16. Completing memory tasks*

17. Imparting knowledge and wisdom*

18. Completing computer tasks*
19. Completing a running race*
20. Looking after children
21. Having a healthy diet
22. Managing staff
23. Taking directions from a supervisor
24. Baking a cake*
25. Recognising the emotion in others' tone of voice*

Note: Items added to Swift et al.'s (2013) questions by the authors are indicated with an asterisk.

2 Supplementary Table 1

Percentage of Participants Who Selected Either "Adults Aged 25", "No Difference", or "Adults Aged 75" as Most Competent in Various Domains, for Each Participant Age Group (Full Table).

Competency domain	Participant age group	Percentage of participants who selected "adults aged 25", "no difference" or "adults aged 75"			Chi-square test of goodness of fit	
		Adults aged 25	No difference	Adults aged 75	X^2	*p*
Solving crossword puzzles	18-30	**32.5**	**43.9**	23.6	$X^2(2, N = 123) = 7.66$	.022
	50-64	23.4	**50.6**	26.0	$X^2(2, N = 154) = 20.94$	< .001
	65+	28.7	**55.2**	16.1	$X^2(2, N = 143) = 34.29$	< .001
	Mean (all ages)	27.9	**50.2**	21.9	$X^2(2, N = 420) = 56.24$	< .001

Being Polite	18-30	18.7	**56.1**	25.2	$X^2(2, N = 123) = 29.46$	< .001
	50-64	1.9	39.6	**58.4**	$X^2(2, N = 154) = 76.46$	< .001
	65+	2.8	39.9	**57.3**	$X^2(2, N = 143) = 66.56$	< .001
	Mean (all ages)	7.1	**44.5**	**48.3**	$X^2(2, N = 420) = 130.56$	< .001
Driving	18-30	**88.6**	11.4	0	$X^2(2, N = 123) = 171.56$	< .001
	50-64	**69.5**	20.8	9.7	$X^2(2, N = 154) = 93.36$	< .001
	65+	**66.4**	25.9	7.7	$X^2(2, N = 143) = 77.59$	< .001
	Mean (all ages)	**74**	19.8	6.2	$X^2(2, N = 420) = 324.90$	< .001
Social Interaction	18-30	**41.5**	**51.2**	7.3	$X^2(2, N = 123) = 39.22$	< .001
	50-64	30.5	**54.5**	14.9	$X^2(2, N = 154) = 36.79$	< .001
	65+	18.2	**67.1**	14.7	$X^2(2, N = 143) = 73.78$	< .001
	Mean (all ages)	29.5	**57.9**	12.6	$X^2(2, N = 420) = 131.67$	< .001
Understanding others' viewpoints	18-30	**43.1**	**35.0**	22.0	$X^2(2, N = 123) = 8.39$	.015
	50-64	14.3	**38.3**	**47.4**	$X^2(2, N = 154) = 27.05$	< .001

	65+	12.6	**38.5**	**49.0**	$X^2(2, N = 143) = 30.06$	< .001
	Mean (all ages)	22.1	**37.4**	**40.5**	$X^2(2, N = 420) = 24.27$	< .001
Learning new skills	18-30	**88.6**	9.8	1.6	$X^2(2, N = 123) = 170.39$	< .001
	50-64	**79.9**	19.5	0.6	$X^2(2, N = 154) = 158.27$	< .001
	65+	**72.7**	24.5	2.8	$X^2(2, N = 143) = 109.94$	< .001
	Mean (all ages)	**80**	18.3	1.7	$X^2(2, N = 420) = 429.10$	< .001
Using the internet	18-30	**91.9**	6.5	1.6	$X^2(2, N = 123) = 190.1$	< .001
	50-64	**88.3**	11.0	0.6	$X^2(2, N = 154) = 211.96$	< .001
	65+	**71.3**	27.3	1.4	$X^2(2, N = 143) = 107.26$	< .001
	Mean (all ages)	**83.6**	15.2	1.2	$X^2(2, N = 420) = 489.44$	< .001
Settling arguments	18-30	11.4	**44.7**	**43.9**	$X^2(2, N = 123) = 26.68$	< .001
	50-64	3.2	32.5	**64.3**	$X^2(2, N = 154) = 86.12$	< .001
	65+	2.1	39.2	**58.7**	$X^2(2, N = 143) = 71.01$	< .001
	Mean (all ages)	5.2	38.3	**56.4**	$X^2(2, N = 420) = 169.81$	< .001

Making financial decisions	18-30	4.9	37.4	**57.7**	$X^2(2, N = 123) = 52.44$	< .001
	50-64	4.5	24.7	**70.8**	$X^2(2, N = 154) = 106.53$	< .001
	65+	7.7	36.4	**55.9**	$X^2(2, N = 143) = 50.53$	< .001
	Mean (all ages)	5.7	32.4	**61.9**	$X^2(2, N = 420) = 199.09$	< .001
Recognising emotions in others' faces	18-30	10.6	**56.1**	33.3	$X^2(2, N = 123) = 38.24$	< .001
	50-64	5.2	**45.5**	**49.4**	$X^2(2, N = 154) = 55.22$	< .001
	65+	4.2	**51.7**	**44.1**	$X^2(2, N = 143) = 55.90$	< .001
	Mean (all ages)	6.4	**50.7**	**42.9**	$X^2(2, N = 420) = 140.70$	< .001
Being creative	18-30	**47.2**	**52.0**	0.8	$X^2(2, N = 123) = 58.98$	< .001
	50-64	32.5	**64.3**	3.2	$X^2(2, N = 154) = 86.12$	< .001
	65+	26.6	**71.3**	2.1	$X^2(2, N = 143) = 105.75$	< .001
	Mean (all ages)	34.8	**63.1**	2.1	$X^2(2, N = 420) = 234.44$	< .001
Understanding how someone is feeling/what they are thinking	18-30	17.1	**59.3**	23.6	$X^2(2, N = 123) = 38.24$	< .001
	50-64	3.2	**42.2**	**54.5**	$X^2(2, N = 154) = 66.25$	< .001

	65+	4.2	**49.7**	**46.2**	$X^2(2, N = 143) = 54.90$	< .001
	Mean (all ages)	7.6	**49.8**	**42.6**	$X^2(2, N = 420) = 128.19$	< .001
Reading for pleasure	18-30	6.5	**44.7**	**48.8**	$X^2(2, N = 123) = 40.15$	< .001
	50-64	3.2	**45.5**	**51.3**	$X^2(2, N = 154) = 63.52$	< .001
	65+	1.4	**45.5**	**53.1**	$X^2(2, N = 143) = 66.90$	< .001
	Mean (all ages)	3.6	**45.2**	**51.2**	$X^2(2, N = 420) = 169.64$	< .001
Completing cognitive tasks (e.g. involving attention, problem-solving, and decision-making)	18-30	**69.1**	28.5	2.4	$X^2(2, N = 123) = 83.32$	< .001
	50-64	**56.5**	37.7	5.8	$X^2(2, N = 154) = 60.56$	< .001
	65+	**53.1**	**40.6**	6.3	$X^2(2, N = 143) = 50.45$	< .001
	Mean (all ages)	**59.0**	36.0	5.0	$X^2(2, N = 420) = 185.33$	< .001
Understanding others' emotional body language	18-30	11.4	**68.3**	20.3	$X^2(2, N = 123) = 69.12$	< .001
	50-64	11.7	**48.7**	**39.6**	$X^2(2, N = 154) = 34.38$	< .001
	65+	4.2	**47.6**	**48.3**	$X^2(2, N = 143) = 54.64$	< .001
	Mean (all ages)	9.0	**54.0**	36.9	$X^2(2, N = 420) = 129.99$	< .001

Completing memory tasks	18-30	**84.6**	13.0	2.4	$X^2(2, N = 123) = 147.27$	< .001
	50-64	**83.8**	13.6	2.6	$X^2(2, N = 154) = 179.\ 08$	< .001
	65+	**77.6**	19.6	2.8	$X^2(2, N = 143) = 132.27$	< .001
	Mean (all ages)	**81.9**	15.5	2.6	$X^2(2, N = 420) = 456.30$	< .001
Imparting knowledge and wisdom	18-30	3.3	16.3	**80.5**	$X^2(2, N = 123) = 126.2$	< .001
	50-64	3.9	7.8	**88.3**	$X^2(2, N = 154) = 209.82$	< .001
	65+	0.7	18.2	**81.1**	$X^2(2, N = 143) = 153.50$	< .001
	Mean (all ages)	2.6	13.8	**83.6**	$X^2(2, N = 420) = 484.900$	< .001
Completing computer tasks	18-30	**90.2**	7.3	2.4	$X^2(2, N = 123) = 179.71$	< .001
	50-64	**82.5**	17.5	0	$X^2(2, N = 154) = 174.62$	< .001
	65+	**70.6**	27.3	2.1	$X^2(2, N = 143) = 103.11$	< .001
	Mean (all ages)	**80.7**	17.9	1.4	$X^2(2, N = 420) = 441.30$	< .001
Completing a running race	18-30	**91.1**	8.1	0.8	$X^2(2, N = 123) = 185.42$	< .001
	50-64	**95.5**	4.5	0	$X^2(2, N = 154) = 268.18$	< .001

	65+	**93.7**	2.8	3.5	$X^2(2, N = 143) = 234.56$	< .001
	Mean (all ages)	**93.6**	5.0	1.4	$X^2(2, N = 420) = 686.61$	< .001
Looking after children	18-30	22.8	**48.8**	28.5	$X^2(2, N = 123) = 13.81$	.001
	50-64	31.2	**45.5**	23.4	$X^2(2, N = 154) = 11.58$	.003
	65+	31.5	**48.3**	20.3	$X^2(2, N = 143) = 17.01$	< .001
	Mean (all ages)	28.8	**47.4**	23.8	$X^2(2, N = 420) = 38.87$	< .001
Having a healthy diet	18-30	19.5	**66.7**	13.8	$X^2(2, N = 123) = 62.1$	< .001
	50-64	7.1	**61.0**	31.8	$X^2(2, N = 154) = 67.26$	< .001
	65+	5.6	**65.0**	29.4	$X^2(2, N = 143) = 76.80$	< .001
	Mean (all ages)	10.2	**64.0**	25.7	$X^2(2, N = 420) = 193.39$	< .001
Managing staff	18-30	26.8	**56.1**	17.1	$X^2(2, N = 123) = 30.44$	< .001
	50-64	16.9	**47.4**	**35.7**	$X^2(2, N = 154) = 21.91$	< .001
	65+	18.2	**44.8**	**37.1**	$X^2(2, N = 143) = 16.04$	< .001
	Mean (all ages)	20.2	**49.0**	30.7	$X^2(2, N = 420) = 53.59$	< .001

Taking directions from a supervisor	18-30	**46.3**	**48.8**	4.9	$X^2(2, N = 123) = 44.93$	< .001
	50-64	**33.1**	**42.2**	24.7	$X^2(2, N = 154) = 7.10$	.029
	65+	23.8	**55.9**	20.3	$X^2(2, N = 143) = 33.16$	< .001
	Mean (all ages)	33.8	**48.8**	17.4	$X^2(2, N = 420) = 62.27$	< .001
Baking a cake	18-30	12.2	**69.1**	18.7	$X^2(2, N = 123) = 71.61$	< .001
	50-64	7.8	**72.7**	19.5	$X^2(2, N = 154) = 110.70$	< .001
	65+	2.8	**69.9**	27.3	$X^2(2, N = 143) = 99.04$	< .001
	Mean (all ages)	7.4	**70.7**	21.9	$X^2(2, N = 420) = 277.39$	< .001
Recognising the emotion in others' tone of voice	18-30	9.8	**69.1**	21.1	$X^2(2, N = 123) = 73.22$	< .001
	50-64	2.6	**55.2**	**42.2**	$X^2(2, N = 154) = 69.36$	< .001
	65+	0	**55.2**	**44.8**	$X^2(2, N = 143) = 73.89$	< .001
	Mean (all ages)	3.8	**59.3**	36.9	$X^2(2, N = 420) = 196.30$	< .001

Notes. Percentages presented in bold are the highest within each participant age group (significance level of $p < .05$). Where the second-highest percentage is statistically equivalent to the highest, that percentage is also bolded.
